# Armed actor interventions in humanitarian and public health crises: examining perspectives of crisis-affected community members

**DOI:** 10.1186/s13031-024-00593-6

**Published:** 2024-04-18

**Authors:** Samuel T. Boland, Alexandria Nylen, Madison Bates, Maria Carinnes Alejandria, Rob Grace, Zein Tayyeb, Adam C. Levine

**Affiliations:** 1 Chatham House, 10 St James’s Square, London, SW1Y 4LE United Kingdom; 2 Brown University, 69 Brown Street, Providence, Rhode Island 02912 USA; 3 Universiti Brunei Darussalem, Jalan Tungku Link, Gadong BE1410 Brunei; 4 Boston University, Commonwealth Ave, Boston, Massachusetts 02215 USA

## Abstract

**Background:**

Despite frequently providing non-military services in times of crisis, little systematic research has examined the perspectives of crisis-affected community members on the role of armed actors responding to humanitarian crises and public health emergencies.

**Methods:**

To address this research gap, 175 interviews were conducted (2020–2021) amongst humanitarian and public health practitioners; armed actors; and crisis-affected community members across three country and four crisis contexts. Specifically, this effort included an Ebola outbreak in the Democratic Republic of the Congo; a refugee crisis on the Jordanian-Syrian border; and a volcanic eruption and COVID-19 outbreak in the Philippines. Data was analysed using grounded theory principles.

**Results:**

Crisis-affected community members held diverse views. Non-state armed groups (NSAGs) and government armed actors were characterised as antagonists by some but supportive by others; gender issues were central to perceptions of armed actors, in ways that were both prejudicing and favourable. Overall perception was most closely linked to armed actor roles rather than the relative amount of conflict in a given area.

**Conclusions:**

Findings nuance the relevant literature characterizing NSAGs as disruptive agents, and also the relevant literature that does not fully consider the nuances of gender and armed actor roles as deeply relevant to crisis-affected community perspectives on armed actors. These findings have important implications for both policy and academic discourse on militarization and localization.

## Background

In the early days of the 2018–2020 Kivu Ebola Virus Disease (Ebola) Epidemic, nearly $9,000 worth of lifesaving drugs were set on fire in the Kalunguta *zone santé* (health zone) in the Democratic Republic of the Congo’s (DRC’s) North Kivu province. At first, there were two interrelated assumptions made by the Ebola response’s coordination body about the incident: that the act was arson, motivated by community distrust of the newly arrived Ebola response; and that the Union of Congolese Patriots (UPLC)—one of the DRC’s dozens of *Mai Mai* militia groups in the east of the country—was responsible. While the first assumption proved accurate, the UPLC denied involvement. In fact, far from acting as a disrupter, the UPLC went on to support the Ebola response, including by guarding the construction of an Ebola transit centre against a later attack (this anecdote is drawn from the study presented in this article).

In addition to providing security, as noted above, the UPLC also reinforced to their combatants and others in the area that Ebola was real, and that the intervening (and often exogenous) Ebola response was essential to their survival. These communication efforts included rallies, and even pro-vaccination publicity stunts. For example, a general of the UPLC was vaccinated in public on a heavily travelled road between the outbreak’s epicentres of Butembo and Beni. The general encouraged passers-by to photograph him and share the images on social media to spread the pro-vaccine message. Maintaining that he nevertheless possessed *gris gris*—a talisman or spell of protection—the general publicly stated that he invited vaccination because, while protecting him against weapons of war, the magic bestowed upon him did not protect against Ebola (*Mai Mai* translates to ‘water water’, wherein water-related charms are believed to protect one from injury and death in conflict) [52]. However, in this instance, the *Mai Mai* leader acknowledged that such protections against weapons did not apply to Ebola, thus recognizing the disease as a public health threat requiring biomedical intervention *vis-à-vis* vaccination (as discussed in this article, other groups were highly disruptive to the Ebola response).

This kind of support by a non-state armed group (NSAG) for a public health emergency response is not usually considered in relevant academic and practitioner discourse found within humanitarian-military relations (HMR),^1^ a field of research and practice focused on engagements between civilian responders and armed actors during crisis response on issues that include armed actors’ involvement in relief delivery, humanitarian access, security, and civilian protection [18, 19]. Indeed, NSAGs have traditionally been characterized in HMR literature as disruptors and threat actors, against which humanitarian and public health actors—as well as crisis-affected community members—must be protected [7, 15, 32]. However, an emerging strand of literature has placed focus on NSAGs as crucial enablers of humanitarian and public responses during times of crisis (e.g., [6, 25]). Indeed, as can be seen in the vignette above, the UPLC had originally (but inaccurately) been suspected of the arson attack in the Kalunguta *zone santé*.

Aside from the risks presented by NSAGs, as above, HMR literature generally focuses on several other key themes and debates. Relevant issues and ideas include, for example, literature typologizing HMR in humanitarian and public health emergency contexts [13, 23, 31, 43, 49, 51]; literature on other risks associated with HMR (including, centrally, the erosion of the Humanitarian Principles of neutrality, independence, impartiality, and humanity) [9, 17, 30, 50]; additional principles and guidelines articulated in global guiding documents intended to inform HMR best practice and overcome HMR challenges [8, 22, 24, 39, 45] and literature on the underlying global political processes of securitization and militarization in response to these kinds of crises [3, 4, 29, 35, 36, 46, 47]. Most of this literature is highly sceptical of HMR, and is primarily concerned with mitigating risks to and challenges for practitioners. For example, the global guiding documents—which, as operational guidance, are unusually accommodating of HMR—nevertheless argue in favour of several principles constraining the breadth and depth of HMR, such as the need for clear distinction between civilian and military actors, and the use of military assets as a ‘last resort’ only. In conflict-affected areas, the documents also caution against any HMR cooperation, and advocate instead for limiting HMR to co-existence only [5].

However, there is little documentation and examination of crisis-affected community members in relation to the role that armed actors play in crisis response. The HMR literature generally assumes that crisis-affected community members have very negative—or at the least, highly fraught—relationships with armed actors, especially in conflict-affected areas (and notably, also generally assumes a positive relationship with humanitarian and other civilian response actors) [18, 19]. Where the documentation and examination of these perspectives does exist, it is usually incidental, or a small component of a wider analysis [11, 34, 41, 42]. Others have documented various other instances of violence by militaries enforcing public health measures during the COVID-19 pandemic [38]. However, this literature does not directly query the perception of crisis-affected community members on the wider array of relevant HMR beyond coercive enforcement. There is also a growing body of literature focused on localization—however, with the notable exception of discourse on issues like NSAG governance at local levels (e.g., [26]), as with other bodies of literature, the discourse on localization is not generally put in conversation with HMR issues specifically.

To address this important research gap, a study was conducted between 2019–2022 to answer the following research question: how do crisis-affected populations experience and perceive the armed actors providing them with assistance in humanitarian and public health emergencies? As the next section will describe, the research process entailed semi-structured qualitative interviews with crisis-affected community members, civilian responders, and armed actors. This article centres the voices and findings from crisis-affected community members specifically. Therein, this article—one of a series drawing on the collected data from four distinct humanitarian crises and public health emergencies—constitutes a formative contribution to the HMR literature.

## Methods

### Site selection

**Fig. 1:**
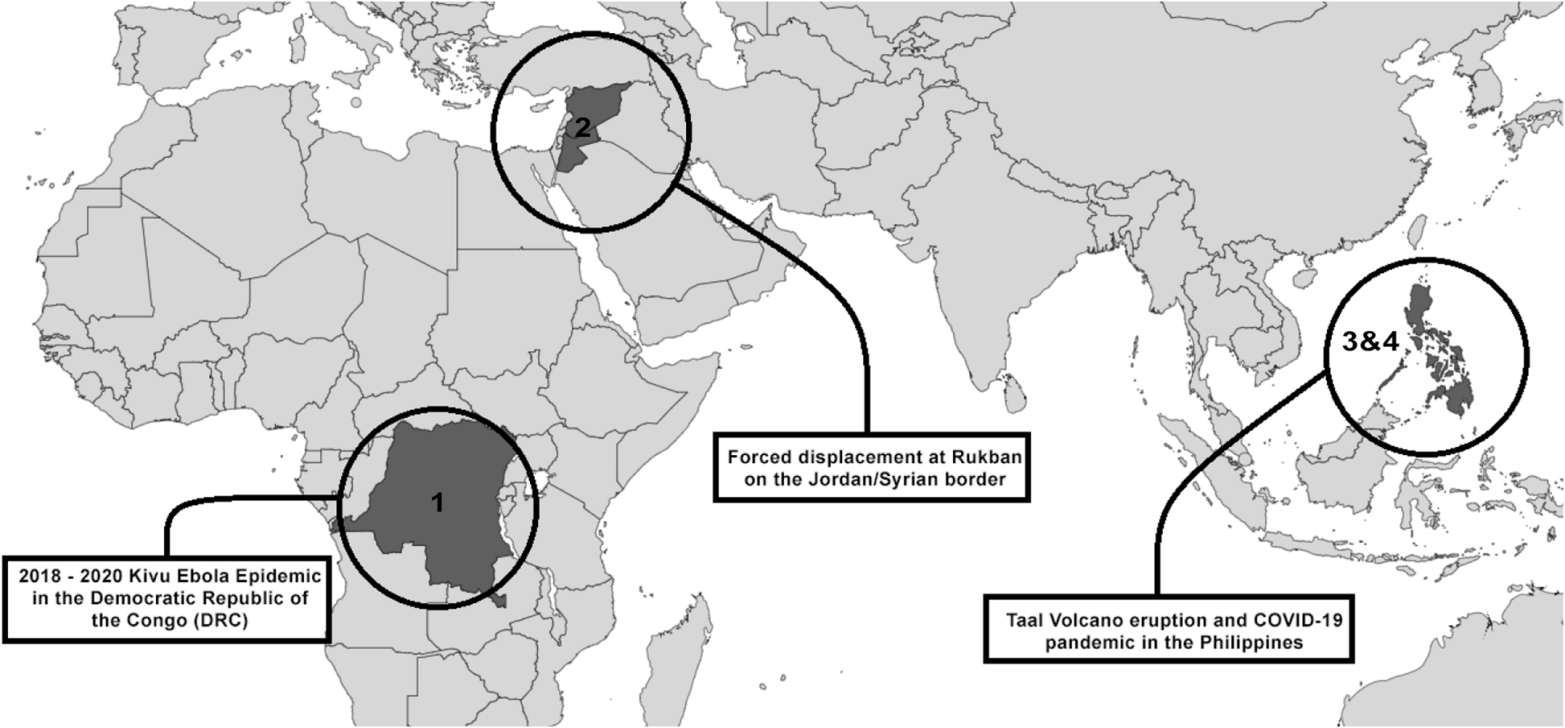
Research sites and crisis contexts

Three research sites were selected for data collection (Fig. 1), representing four crisis contexts. These sites were selected due to their significant diversity and relevance to the research project, and also due to the principal researchers’ familiarity with (and therefore ability to access) the different contexts. Each is described in turn.

#### Democratic Republic of the Congo

The 2018–2020 Kivu Ebola Epidemic was selected to investigate HMR during an epidemic response in an area facing protracted internal conflict. In-country site selection traced the course of the outbreak from its origins in Mangina and its spread to the subsequent epicentres of Beni and Butembo. Other relevant locations within the region were chosen for study, including the North Kivu provincial capital of Goma (where the Ebola response’s national coordination was situated for much of the outbreak, and also where the headquarters of the United Nations (UN) Organization Stabilization Mission in the DRC (MONUSCO) is situated).

Relevant armed actors include *Mai Mai* groups, as well as other NSAGs including highly violent terrorist groups such as the Allied Democratic Forces (ADF). The former does not refer to any particular movement but rather a broad variety of groups (some estimates place the number at more than 100) that ostensibly protect communities—though many are associated with looting, cattle rustling, and banditry, amongst other offences [28]. Given their number, they range in size, modes of governance, and objective, and accordingly, have diverse relationships with local populations [28]. The ADF, meanwhile, is a relatively large NSAG best characterised as a radical terrorist organisation that indiscriminately targets civilians across a broad geography of North Kivu with the stated objective of creating an Islamic polity [55]. Other relevant armed actors include governmental forces—both the army (FARDC) and police (PNC)—and also MONUSCO. Each of these groups has a complex relationship with Ebola-affected populations, ranging from highly fraught (e.g., the FARDC who are widely documented to have committed various human rights abuses) to supportive (addressing the history of these relationships in detail is beyond the scope of this article, but has been examined elsewhere) [28].

#### Rukban (on the Syria-Jordan border)

This site allowed for an examination of HMR in a geopolitically charged setting of significant population displacement, and where humanitarian aid workers confronted serious security risks. Research at this site centred around the ongoing crisis in Rukban (2014–), to where tens of thousands of Syrians were displaced and became stranded, unable to enter Jordan. This specific location was chosen due to the extreme restrictions on humanitarian access and the intensive engagement and negotiation between humanitarian and armed actors that was required to navigate the context. Crisis-affected community member interviews were not conducted in Rukban itself (as it remains inaccessible), and were instead conducted with individuals who had been transferred from Rukban to the Zaatari and Azraq refugee camps deeper within Jordan (this may present research limitations reflected on later in the Methods section). Relevant armed actors include the Syrian army—with significantly negative relations to crisis-affected populations—and the Jordanian Armed Forces (JAF) controlling border access into Rukban, who were not party to the conflict but were nevertheless key actors in the humanitarian crisis.

#### Philippines

This site allowed for an examination of HMR during the response to a locally led natural hazard (Taal Volcano eruption, 2020) as well as a pandemic (COVID-19, 2020–) in a context dominated by counter-terrorism concerns. Four sites were selected to represent the experiences of crisis-affected community members. Specifically, the cities of Batangas and Cavite were selected due to their proximity to the Taal Volcano. Quezon City was selected as a key site for understanding the pandemic response, as the area with the highest positivity rate in the country at the time of data collection. Finally, Rizal Province was selected as a representative of the expanded and highly restrictive quarantine bubble that was assigned by the country’s Inter-Agency [COVID-19] Task Force (IATF). Relevant armed actors include the national army, as well as the country’s armed police.

### Subject selection

Interviewees were selected from three overarching groups: humanitarian and public health actors, armed actors, and crisis-affected community members (defined in Table 1). These groups were selected due to their centrality to HMR (*i.e.*, the former two groups comprise HMR, while the latter group is that most directly affected by the crisis at hand). Note, in this article, both state and non-state armed groups are collapsed into the category of ‘armed actor’. As with all research groupings, this grouping contains significant diversity. In the data, where a given armed actor is being discussed, it is referenced by name. Across respondent groupings and data collection sites, a total of 175 interviews were conducted (Table 1).

**Table 1:** Defining respondent groupings in this article (source: author)

#	Actor grouping	Definition as used in this article	Research site	Total # per site	Total #
**1**	**Humanitarian and public health actor**	Any civilian actor formally responding to the crisis at hand—*e.g.*, UN agencies, (international) non-governmental organizations ((I)NGOs), civil society organizations (CSOs), *et cetera*	Jordan	25	62
			Philippines	17	
			DRC	20	
**2**	**Armed actor**	Any member of a state or non-state armed group (including, where armed, police and gendarmerie)	Jordan	–	20
			Philippines	12	
			DRC	8	
**3**	**Crisis-affected community member**	Anyone affected by the crisis at hand, who is not formally a member of the response and does not fall into one of the other actor groupings	Jordan	19	93
			Philippines	35	
			DRC	39	
-	**Total**	-	Jordan	44	175
			Philippines	64	
			DRC	67	

As part of a wider multi-year study, this article focuses specifically on the examination of crisis-affected community member perspectives (the other perspectives are therefore not systematically assessed in this article, though where relevant, are reflected on). So as to improve access and overcome language barriers, all community member interviews were conducted by trained and supervised local interviewers. Local interviewers were also responsible for identifying respondents, with a focus on diversifying: age (though limited to 18 and above); gender; religion; and other demographic factors relevant to the context. Therein, eligibility criteria was purposefully broad, to include any crisis-affected adult. Data collection continued until saturation was met. In all cases, supervision of local interviewers was provided by the principal researchers. This included pre-interview training; a post-interview review of an initial tranche of transcripts; and post-interview discussions to discuss challenges, opportunities, and possible new research leads for subsequent investigation. All interviews conducted in DRC and those related to the Rukban case were conducted in person; in the Philippines case, most were conducted by telephone due to COVID-19 lockdown restrictions (discussed further in the Limitations section).

Interviewers used similar interview techniques in all contexts. Interviews were 1:1; open-ended in nature (i.e., interviewers were free to be guided by the discussion at hand); and lasted approximately one hour each. Interview guides—which were adapted according to the crisis context—were used based on key considerations from the literature, and were also iteratively elaborated on during data collection as new lines of investigation were raised. Saturation was considered throughout. Interviews were audio recorded with interviewee consent, translated into English, and transcribed in an anonymized manner.

### Analysis

All interviews were transcribed and input into NVivo or MAXQDA for coding. Coding—which was conducted by the principal researchers—was primarily inductive (i.e., themes and patterns were derived from the data, following the principles of grounded theory). In grounded theory, nodes are created as new themes arise in the data; from which abstract categories are constructed inductively and social processes discovered [37]. In other words, each line of each transcript was examined for concepts and key phrases which were (sub-)categorised—where relevant, against multiple nodes—to iteratively develop a codebook. The codebook was thus elaborated on throughout the data coding process. Upon completion of the coding process, the codebook was re-evaluated, and nodes (dis)aggregated where appropriate.

Once all interviews had been coded, memos were produced using principles of framework analysis (e.g., key themes were charted and mapped within and between the research groupings). Memos were then thematically compared in a series of extended discussions by the research team, so as to identify and resolve any incongruence or gap; to avoid siloing; and to develop a framework comprised of the relationship between overarching themes (derived inductively) that are presented in this article (i.e., following this methodological process identified the key themes reflected on in this article).

### Ethics and funding

Ethics approval was requested and granted from the Brown University Institutional Review Board (Protocol 1,912,002,591), *Université de Kinshasa École de Santé Publique Comité D’Éthique* in the DRC (protocol ESP/CE/17/2020 and ESP/CE/17/2021), and the Philippine Social Science Council (Protocol CF-20–02). Local approval was not required in Jordan. Best practice for data collection, storage, and presentation was followed throughout.

Funding was provided by the United States (US) State Department Bureau of Population, Refugees, and Migration (PRM), which had no role in the design of the research study, the collection or analysis of data, or the findings presented in this article. Therefore, statements within this article should not be taken to necessarily reflect the position(s) of the US State Department.

### Limitations

There are several plausible limitations to this research study. As a multi-site project, site selection presents a number of research strengths—*i.e.*, there is the ability to compare and contrast perspectives from multiple contexts, and therefore to robustly test and nuance the findings from any one site. However, site selection also presented challenges that possibly limit the generalizability of this research project. For example, parts of North Kivu and Ituri provinces in DRC were inaccessible to in-country research staff due to ongoing insecurity. While this did not prevent access to all research groupings, it did limit access to some crisis-affected communities. Due to this very insecurity, it is plausible that individuals in these areas hold different views on armed actors than those documented and presented in this article. In Jordan, accessing Rukban itself was not possible (nor was it possible for the research team to access other parts of Syria which would have represented invaluable sites for data collection). This was mitigated by conducting interviews with displaced persons who were prior inhabitants of Rukban, but had since moved on to other areas which were accessible. However, this did mean interviewees recollected experiences over the past several years, which introduces the possibility of recall bias (further, the individuals were therefore physically located in Jordan. While many expressed concerns about their living conditions in the Azraq and Zaatari camps, it is nevertheless plausible that for this protection, the respondents had more positive associations with the Jordanian state—and by extension, the JAF—than they would otherwise). Site selection limitations were further mitigated by the overall large number of crisis-affected community members that were interviewed (*n* = 93) and efforts to reach saturation, which helped ensure the documentation of diverse perspectives.

Subject selection also presents limitations to study generalisability. Of particular note was the difficulty accessing armed actors. For example: in Jordan, military personnel were approached for interview, but none consented, and in the DRC, it was not deemed safe for in-country research staff to seek interviews with members of the FARDC (though requests were nevertheless made by remote research staff through official channels). As key stakeholders, the inability to include these actors remains a limitation of the broader study. There is also a gender skew in the dataset, in particular amongst the armed actor grouping in which the overwhelming proportion were male. To an extent, this reflects a gender skew inherent to the grouping. However, provided the relevance of gender to HMR discourse (as discussed in this article), this nevertheless represents a research limitation. However, this article focuses on the perspectives of crisis-affected community members, which mitigates this specific limitation. These limitations were further mitigated through the overall large number of interviews across all research sites and respondent types, and the overall effort to reach saturation.

## Findings

Both across and within each of the examined cases, crisis-affected community members varied greatly in their perspectives on armed actors, with characterizations ranging from gratitude and acceptance to condemnation and repudiation. This section presents three themes—derived inductively from the data—on how crisis-affected community members perceived and interacted with armed actors in the examined crisis contexts. First, this section discusses crisis-affected community members’ perceptions of armed actors in relation to the different roles that armed actors played in the response. Second, the section discusses perceptions of the role of NSAGs in particular. Finally, gender-based dynamics of HMR are discussed, given their import for how crisis-affected community members perceived and received armed actors in the examined cases.

### Function over form

Crisis-affected community members’ perspectives of armed actors in the examined cases was heavily influenced by the content of the role being performed. Generally speaking, across the examined cases, respondents felt that armed actors’ traditional security roles and the provision of direct aid and other services were the least inconsistent with expectations (whereas, as discussed below, enforcement and public health roles were more controversial). In Rukban, for example—as detailed in the later section on gender—respondents were grateful for the protection they associated with the JAF, and also spoke positively about the JAF’s provision (albeit limited) of food and healthcare to those arriving at the border.

Generally positive associations with armed actors were also expressed by crisis-affected community members recalling the response to the Taal volcano eruption in the Philippines, during which the Filipino national army played a significant role aiding evacuations and running centres in which displaced persons could receive food, shelter, and other assistance. One respondent recalled:

*I was very happy because I knew we would be receiving help from other people. We were glad to know that we had a support system in these rescue operations. The military is not just about chaos. I believe that the military helps for humanitarian reasons like rescuing and relief operations.*

Nevertheless, for their role contributing to (in)security on the one hand, this respondent saw armed actors as agents of ‘chaos’; for their provision of direct aid and other services during the response to a disaster triggered by natural hazard, this respondent saw armed actors as agents of humanitarian relief. The former association did not preclude the respondent holding what appears to be a dissonant view, if and when the armed actor was providing aid. In other words, the respondent was sensitive to context and function, rather than understanding or seeing armed actors as of single purpose and effect. Across the examined cases, crisis-affected community members were generally understanding (as distinct from accepting) of armed actors’ security functions, and also receptive to their provision of direct aid and other services where relevant. Indeed, no respondent stated an inherent issue with the latter role.

However, in the DRC and the Philippines’ COVID-19 case, crisis-affected community members expressed a diverse (and more often negative) perspective on the role of armed actors. This was especially the case when armed actor roles included enforcement functions. In the DRC, for example, a small majority of crisis-affected community members expressed confusion and consternation when recalling armed actors’ escort roles—wherein armed actors escorted civilian Ebola responders to not only protect them, but also to ensure their access to certain areas. One respondent explained (in a way that was illustrative of many other crisis-affected community member perspectives):

*First of all, the disease itself, you cannot say that a nurse must be accompanied by a military or a police officer. Absolutely not. This is what was* [making people] *question, ‘Is this really this disease, where the nurses walk around with the army and the police, is it really a disease? Is it not a political thing?’ So, all the questions* [that] *were circulating in the heads of the population* [were]* because of the presence of the army that accompanied the nurses in different environments.*

In the Philippines’ Taal and Rukban cases examined above, the function of armed actors was understood, if not appreciated. In this public health instance, however, the respondent felt the armed actors’ role was deeply incongruous with the crisis at hand. This, in turn, helped construe the outbreak as related to political machinations (which was facilitated in no small way by the decision to disenfranchise eastern Congo from the 2018 elections due to alleged Ebola-related risks).

Across cases, these sentiments were widely reported by crisis-affected community members when considering armed actors’ enforcement roles. In the Philippines’ COVID-19 case, for example, a respondent made the following statement:

[The armed actors] *were roaming around the area. It wasn’t giving any sense of relief, aside from additional fear. If they were from the health sector, I would totally understand. There were times that they were roaming around armed with weapons. You don’t know, what is it for? Are they going to attack someone? Were they having* [military and security] *operations?*

These various respondents—as illustrative of a majority of crisis-affected community members—associated armed actors so strongly with more typical security and direct aid functions, that enforcement roles led to suspicion that ulterior motives were at play. It should be noted that there was important dissent on this point amongst a small but meaningful minority of crisis-affected community members in both the DRC and Philippines’ COVID-19 case, who felt that militarized enforcement of public health measures ensured strict compliance in a way that was both effective and valuable.

A related theme—one that was particularly pertinent in DRC—was crisis-affected community members’ consternation that enforcement roles distracted ‘benevolent’ armed actors from helping protect communities from ‘malevolent’ ones. For example, when asked about MONUSCO’s role supporting the Ebola response, one respondent stated:

*It's true, they have a goal, yes, they can help us. But there is a mission to stabilize* [things] *here at home… And for me, personally, I won’t refuse if they do other ordinary work, but, on* [the] *condition that they have stabilized*… *Even though they helped us with* [the Ebola] *disease,* [there remains] *the one who forcibly kills me while I* [work] *my fields.*

When asked to further clarify the appropriate role of MONUSCO and government troops, the respondent continued:

*Their job is to protect people and their property. So, when we see* [they are] *not following up* [and protecting people]*, confidence starts to drop. But, it is our army. We cannot trust elsewhere* [i.e., exogenous armed actors].* But, it is because of them that the confidence of the population in them disappears, since they are not doing their job as planned.*

In other words, this respondent accepted armed actors’ enforcement and public health roles, but only ‘on [the] condition’ that security was first maintained. Without first ‘doing their job as planned’, ‘the confidence… of the population… disappears’—which, as corroborated by a number of other crisis-affected community members, further contributed to allegations of ulterior political and financial motives.

Table 2 presents a typology that summarises these key roles (as reflected in the data, these roles can—and sometimes did—occur simultaneously, and/or were sometimes interrelated).

**Table 2:** Examples of armed actor roles in the examined cases in which direct interaction with crisis-affected communities might occur (Source: author)

	**(In)security**	**Enforcement**	**Direct aid and other services**
*Role of armed actor*	Causing or risking violence against crisis-affected community members through conflict or protecting crisis-affected community members from insecurity	Policing and other law enforcement functions to coerce crisis-affected community members into abiding by certain measures	Delivering direct aid and other forms of assistance to crisis-affected community members
Illustrative example drawn from interviews	Various NSAG attacks on crisis-affected communities in eastern DRC	PNP enforcing crisis-affected community lockdowns by staffing COVID-19 quarantine checkpoints	JAF medical services provided to crisis-affected community members in and around the Rukban camp

### Perceptions of NSAGs

NSAGs were highly relevant to each response,but played very different roles both within and across all three cases. In some circumstances, NSAGs were antagonistic to the response at hand, such as the violent extremist ADF group in DRC. In other circumstances, however, NSAGs proactively supported humanitarian responses—and on occasion, even performed humanitarian-type activities themselves. Therefore, the involvement of NSAGs was a key factor influencing crisis-affected community members’ perspectives of armed actors more generally in the humanitarian and public health crises that were examined.

In the DRC case, crisis-affected community member respondents generally expressed strong opinions about state and non-state armed actors’ role in the Ebola response. However, there was no obvious consensus to this perspective: many spoke openly in support of armed actor intervention, many spoke against it, and many had differing opinions depending on the armed actor in question. Some respondents, for example, reported that local *Mai Mai* militia groups played positive roles in public health outreach and communication, with one stating these groups were “*especially spirited*” towards such activities when cases arose in their territories. For example, one respondent stated:

*There was the* [militia’s] *commander, who had directly started to raise awareness and tell people that the disease really exists. He said, ‘Here at home we refused that the disease does not exist. Now people are dying’... So, the gunmen who are not in the government have also helped make people* [i.e., the general public] *understand that the disease exists. I have seen it with my own eyes, these armed people carrying out good outreach. People really listened to them.*

In other words, the militia commander worked proactively to raise awareness of Ebola, and to convince crisis-affected community members to take the outbreak seriously.

Another crisis-affected community member reported observing similar behaviour on the part of a different militia leader, who leveraged local networks for public health outreach:

*One day, their* [the militia] *commander even sensitized us here in this room where we are seated. He told us, ‘The disease exists, don't be obstinate! This disease kills the proud, and we have to get to work to eradicate it’… There was indeed such a case in the village of Mahohe. Almost 9 people from the same family had died of Ebola. There was great resistance in this family.* [The militia commander] *went there and convinced them* [not to resist the Ebola response]*. If it weren't for his actions in those days, Ebola would still be in Mahohe. He convinced the mothers who sell pineapples* [by the road] *to tell them that the disease is indeed there, and that we must protect ourselves.*

As indicated in this quote, the DRC case demonstrates the diversity of how NSAGs might interact with a response, and how crisis-affected community members perceive these different interactions. For example, one respondent simply stated: “*it’s only the ADF that bothers us*”.

Indeed, due partially to the violent actions of the ADF, some respondents spoke very positively about the protection afforded them by other NSAGs. The UPLC, for example, reportedly protected response staff, supplies, and buildings against the ADF:

*Those from the UPLC supported the response and avoided* [causing] *any damage. For example, the ETC* [Ebola Treatment Centre]*, when it was built here* [in Kalunguta]*, people thought it was a slaughterhouse to kill the people inside. The UPLC Mai Mai have done everything to keep it* [safe]*. It is thanks to them that this ETC is still there, and it is thanks to them that Ebola was quickly eradicated here.*

In addition to this protective function, crisis-affected community members also praised certain NSAGs for the role they reportedly played in facilitating access for Ebola response teams. For example, one respondent stated:

*I can say that our peripheral roads are maintained and the city are clean thanks to the Mai Mai… They tell the men of such and such a village to do the work, so that the road is well maintained. I remember there was a bridge somewhere. They had done everything to maintain the bridge and let vehicles pass through it without problem.*

Notably, the support that crisis-affected community members expressed towards some NSAGs was not necessarily extended to the FARDC and their involvement in the Ebola response.

For example, reflecting on the FARDC’s role providing armed escorts to public health responders, one crisis-affected community member stated:

*When we started to see the soldiers, then the doubt started to set in. We said to ourselves, ‘Who is this patient that we have to look for with a gun?’.*

Another echoed similar hesitation and consternation for the role of the FARDC:

*Usually, when the population begins to demonstrate… the army has always intervened, and this has always created a kind of opposition between the army and the population. Obviously, during the response, the population also asked themselves many questions. ‘Why are armed people also intervening in the response? Do they also want to force us to die?’… For some people, seeing the army in the activities of the response, they interpreted that it is a way to eliminate us. Because we do not want to accept them, and they will eliminate us by force.*

To an extent, then, the historically fraught relationship between the FARDC (and the government more generally) and the population of North Kivu and Ituri provinces meant that NSAGs were usually received more positively than the FARDC. Hence, another respondent simply stated that “*a soldier or policeman is always bad”.*

### Gender dimensions of HMR

Gender was a key factor influencing crisis-affected community members’ perspectives of armed actors’ presence and role in response to the examined humanitarian crises and public health emergencies.

In the Rukban case, for example, crisis-affected community members generally felt either neutral or positive towards the Jordanian Armed Forces (JAF). The JAF were situated at the border between Jordan and Syria, and were, in many ways, gatekeepers to the Rukban camp. Therefore, during the process of displacement from Syria, the JAF were the first visible evidence of having reached the border. Respondents consistently noted relief and gratitude for the safety and security they therefore associated with the JAF—including for reasons specifically related to the large number of female refugees. For example, one crisis-affected community member recalled:

[The JAF’s] *role at the camp was to make sure it was secure. When we arrived, they told us that any troublemakers would face consequences,* [and that troublemakers] *may not be allowed to enter. So, they kept us in check. We were very happy with their role, especially since there were women and children,* [and]* widows without protection.*

In other words, this respondent felt that the presence of the JAF alleviated particular risks facing female refugees from other displaced persons (‘troublemakers’) that resulted from the absence of male family members who would otherwise provide ‘protection’.

Other female respondents specifically highlighted differential—and preferential—treatment they felt they experienced from the JAF due to their gender. For example, one recalled arriving at the border, and waiting in a kind of no-man’s-land, unsure of where to go as the entrance to the camp was closed at the time. Only those in need of urgent medical attention were being permitted entrance. She waited.

*Then, at night, a few tanks approached, as well as an ambulance and some* [military] *physicians.* [I had a small injury on my foot], s*o I went to have them look at my toe, and one of them said it needs to be amputated. He said* [to me privately]*, ‘There’s* [actually] *nothing wrong with you… don’t be afraid… but I’m saying this to find a way to let you in* [to the camp]*’… I will never forget this. It was very humane of him… The women, just us the women, were placed in the ambulance… Now we were special… and we* [were therefore able to] *enter the first checkpoint* [into the camp]*.*

In this instance, the respondent felt that the JAF granted them access to the relative safety of Rukban specifically because they were women. Indeed, in this instance, the ‘humane’ treatment—purposefully misassigning her ailment as a medical emergency, so that she was ‘special’ and was therefore permitted access into the camp—was extended only to the other women and their children waiting at the border.

However, while treatment by JAF personnel was generally remembered as being quite positive (other examples included JAF personnel carrying refugees’ luggage and assisting the elderly), it was not necessarily consistent. One female respondent and mother, for example, recalled how JAF personnel.

*…registered her* [eldest daughter’s] *information. They took pictures with her, and have these saved to this day. They asked me if I could leave her with them! I said, ‘No way!’. I left Syria primarily for my children. I’d never leave her.*

In her interview, the respondent characterized this as a ‘bad apple’ incident, and as with other respondents, generally associated the JAF with protection. Indeed, in the Rukban context, respondents’ examples of this kind of predatory behaviour by armed actors were very few. However, it nevertheless evidences a particular risk faced by women in such insecure environments—even in ones where military personnel were almost always felt to be protectors.

This notion was echoed strongly in the Philippines, where during the COVID-19 response, the (armed) Philippines National Police (PNP) operated checkpoints (as raised by respondents, the PNP’s armaments and tactical camouflage made their personnel difficult-if-not-impossible to distinguish from members of the Armed Forces of the Philippines). These checkpoints were to enforce lockdowns that—in theory—were in place to protect communities.

However, several crisis-affected community members recalled how these armed checkpoints presented opportunities for gendered coercion including sexual harassment and assault. Second-hand—though as corroborated by numerous sources in the grey literature [16, 53, 56], such as those referring to quarantine cordon ‘sex-for-pass’ schemes in which multiple rape victims were reported on a daily basis—one respondent said:

*The fact that you hear reports about these guys using their authorities to rape women at the checkpoint, that’s the thing that gets me emotional.*

Indeed, for many female respondents, interactions at checkpoints became inherently gendered experiences, provided that PNP personnel—as with most armed forces in all contexts examined in this study—were overwhelmingly male. Another female respondent recalled their first-hand experiences navigating the checkpoints:

*Then this military person, when I rolled down the window, came out as aggressive. It was like, direct interaction. And that bothered me. Women, especially women of marginalized groups, were being harassed, so I guess there was this notion in my head that when they stopped me, I should have my defences out. I was like, for me, there was a bit of anxiety.*

In this case, therefore, interaction with armed actors became highly gendered experiences, in a way that included confrontation and harassment, and which resulted in defensiveness and anxiety. Far from being understood as protectors, in the Philippines’ COVID-19 case, armed actors were generally performing an enforcement role, for which they were feared.

In the DRC case, no study respondents in the crisis-affected community member category concretely recalled gender-based violence or other gender-based interactions with armed actors..However, sexual violence by armed actors is rife in eastern Congo, with widespread documented evidence of these abuses by NSAGs, FARDC, PNC, and MONUSCO personnel. It should also be noted that civilian Ebola responders have been widely reported to have perpetrated sexual abuses as well) [40, 44, 48, 57, 58].

## Discussion

This study sought to examine the views of three research groupings across four crisis contexts, specifically highlighting the perspectives of crisis-affected community members as related to the role of armed actors.

Findings indicate important divergences in the perspectives of this research grouping with others—in particular, humanitarian and public health actors (which were assessed and described in other research outputs) [1]. This finding was most evident when considering the role and perception of NSAGs. Without exception, in the relevant case study (DRC), NSAGs were characterized by humanitarian and public health actors as highly disruptive, threatening, and menacing [1]. The extremely and indiscriminately violent ADF, for example, was always considered by all respondents as an adversary and threat (noting that no member of the ADF was interviewed for this research project, which is a limitation of this article). 

However, crisis-affected community members were very diverse in their characterization of *Mai Mai* NSAGs, with approximately half casting them in a positive light—including for their roles supporting the Ebola response. As reflected in the data, these NSAGs circumstantially supported the response in three key functions: public health education and community vaccine outreach, protection of response staff and infrastructure against attack by other NSAGs, and infrastructure maintenance. This finding is in sharp contrast to the perspectives of humanitarian and public health actor respondents, who solely characterized NSAGs as malevolent and harmful, as above [1]. Indeed, in the prevailing literature, the prevalence of NSAGs was one of the key reasons for the heavy securitization of the response, including the systematic use of armed escorts and co-location of militarised security at offices and residences (going against global guidance and the concept of acceptance as a security management approach) [12, 21, 24]. Numerous respondents in the community and civilian responder research grouping referenced these concerns, with many citing them as justification for the controversial use of armed escorts throughout the 2018–2020 Kivu Ebola Epidemic (which—as referenced by a number of humanitarian and public health actor respondents whose statements are examined in other articles—also included some organizations’ highly controversial cash payments to the national army for these and other services). This securitization had the unintended effect of alienating crisis-affected community members from the response, since locals reported high levels of distrust towards the police and military due to historical disenfranchisement and harassment.

In this study, however, some crisis-affected community members placed their trust in the very NSAGs that Ebola responders worked to fortify themselves against. To an extent, this trust in local militias could be connected to a desire for greater localisation by crisis-affected community members. For example, while the generally exogenous approach taken by international public health experts sometimes estranged local populations, *Mai Mai* militias were embedded in their communities, and may indeed therefore have closer political alignments than the national army headquartered in a capital city just over a thousand miles away). This inference corresponds with research conducted in other contexts—for example, that examining the legitimacy of the Taliban in Afghanistan and the notion that, in most areas under their control, the Taliban are themselves local people [25, 26]. It is also an important axis on recent research proposing a new HMR typology [20]. As highlighted in the data, that *Mai Mai* militia were usually grounded within their respective communities allowed many to support very localized response activities, such as recruiting mothers and petty traders to inform their customers about the importance of getting vaccinated against Ebola.

Importantly, community perceptions of NSAGs should not necessarily be taken to indicate the motivations that such groups might have for participating in the response to a humanitarian or public health crisis. That is, how an NSAG interacts with such a crisis response could depend on how that specific group defines its own interests and goals and how it self-organises (see, e.g., [33]). For example, it is plausible that *Mai Mai* groups found utility in the increased perception of legitimacy for their actions supporting the Ebola response (civilian support has been identified elsewhere as centrally important to rebel success) [2]. Regardless, that does not necessarily mean the protection and support of civilian populations is antithetical to a groups’ objectives especially when there is political alignment with them. Hence, in other related contexts, groups like Geneva Call work to guide NSAGs on issues of civilian protection [14]. Relatedly, communities themselves have been found by scholars to have a degree of agency in negotiating their relationships with NSAGs [27], which they may not necessarily have with other actor groups. Contrarily, the ADF’s organizational goals include resisting all exogenous actors through the use of violence [10]. Therefore, such an organization could plausibly lose legitimacy with its radical base if it was perceived to be supportive of a crisis response [2, 33]—especially one that was not only government sanctioned, but also supported by their adversary MONUSCO.

In addition to the divergence of perspectives with humanitarian and public health actors, perspectives of crisis-affected community members also diverge on a number of issues that comprise a significant body of HMR literature. The global civil-military guiding documents, for example, advocate for selective cooperation between humanitarian and military actors during times of peace, but mere co-existence in times of conflict (i.e., without a working relationship, with a two-dimensional spectrum of closeness between these nodes). In principle, this approach aims to ensure that—in politically fraught contexts in particular—distinction is maintained between the civilian and armed actors [8, 22, 24, 39, 45]. This approach, posit the global guiding documents, supposedly helps to prevent the politicization of aid. Likewise, the Humanitarian Principles of neutrality, independence, and impartiality are intended to clearly delineate civilian and military roles, so as to avoid conflation of these actors by the general public [9, 17, 30, 50].

In the four examined cases, however, the level of conflict had little relevance to how crisis-affected community members perceived and received armed actors. In the Rukban case, for example, the level of conflict was extremely high, but respondents were consistently appreciative of the support being provided by the JAF. Of course, the JAF were not party to the conflict, which presumably affected crisis-affected community perspectives of the degree of protection they had once being received by the JAF. Nevertheless, it was the very extent of conflict in Syria that resulted in the degree of gratitude towards the JAF that was expressed by crisis-affected community members—whereas, plausibly, were there less extreme conflict in Syria, the presence and perceived protection of the JAF would have been less significant. Put simply, the main factor affecting respondents’ perspective was that one armed group created very significant insecurity, while the other mitigated it. In DRC, where low-level conflict was entrenched and all armed actors to some extent were perceived to create conditions of insecurity, respondents were very mixed in their receptivity to the support of armed actors. To some, the level of conflict meant the presence and role of armed actors was unacceptable—but, to an almost equal number of others, the level of conflict justified armed actors’ support, including by NSAGs, as discussed above. Further, in identifying armed actors as improper public health ones, respondents tacitly but clearly delineated them from civilian responders. This is despite the robust civil-military cooperation and interactivity throughout the 2018–2020 Kivu Ebola Epidemic, for which the literature would generally predict problematic association by members of the general public [18, 19]. In the Philippines too—the most peaceful of the examined contexts—crisis-affected communities were extremely diverse in their relative receptivity to armed actor roles: in the Taal volcano case, the support of armed actors was relatively uncontroversial and generally accepted; in the COVID-19 case, the support was highly controversial and generally characterized as anathema to the overall mission of protecting crisis-affected communities (noting that natural disasters are fairly common in the Philippines, whereas COVID-19 was novel and therefore not well understood by many).

In all cases, therefore, the level of conflict had little relevance to whether armed actors were accepted by crisis-affected communities, or whether crisis-affected communities politicized the support being provided by other civilian responders. Rather, regardless of area context, it was the content of armed actors’ roles that was of primary relevance: (in)security functions were considered conventional; direct aid and other services were generally appreciated (or even embraced); and enforcement roles were generally characterized as confusing at best, and ripe for abuse at worst. Relatedly, whether armed actors and their assets were used as a ‘last resort’—a defining principle of civil-military cooperation in response to humanitarian crises, especially in areas affected by conflict—was of little concern to crisis-affected communities [8, 24, 39, 45].

While the diversity of these perspectives may seem unsurprising to those trained in anthropology or sociology—disciplines that generally argue for how communities are comprised of a number of diverse, pluralistic, multifarious, and complex positionalities (see, e.g., [26])—this is a very disruptive finding for a wealth of HMR discourse, and in particular, global guidance. This is because the global guiding documents—which underly a considerable amount of civil-military ‘best practice’—classify the desired breadth and depth of acceptable HMR according to the level of conflict prevalent in particular contexts. In the examined cases, area context could not predict the extent to which crisis-affected communities were or were not concerned by the presence or role of armed actors supporting humanitarian and public health endeavours. That is not to say that these perspectives were not sensitive to context, but rather, that the perspective of crisis-affected community members on armed actor roles cannot be assumed or generalized. Conceivably, therefore, a set of key considerations or a framework of core principles—alongside robust social science data collection and analysis—would be most appropriate for tailoring HMR to a given crisis. This should include—but not be limited to—consideration of factors raised in the data such as military and NSAG roles, as well as gender, alongside robust protections such as those delineated in the Humanitarian Charter and Minimum Standards in Humanitarian Response (‘the Sphere Handbook’) [54]. Regardless of the diversity and complexity of these issues, what is unambiguous is the need to better document the perspectives of members of crisis-affected communities on HMR and other civil-military themes, so as to appropriately contextualize any HMR considerations being made during ongoing and future crises.

## Conclusion

Although community members are most directly affected by a given humanitarian or public health emergency, there is a lack of research that systematically documents and examines their perspectives on the role of responding armed actors. Their voices, in short, are persistently un(der)heard. This is a significant and plausibly dangerous gap found in much of the HMR literature, especially global guiding documents such as the Oslo Guidelines. Accordingly, this study systematically documented and examined these important perspectives (as well as those of crisis responders, so as to compare and contrast them). Crisis-affected communities were found to hold diverse perspectives on several key HMR themes, including the circumstantial endorsement of NSAG support. Gender and the specific role of armed actors were also found to be important factors influencing the overall acceptance of armed actor assistance. These findings nuance—and at-times challenges—HMR literature that often assumes a contentious relationship between crisis-affected community members and armed actors, particularly as assumptions dependent on the degree of area conflict. In DRC, for example, the degree of area conflict was very high, but perceptions of armed actors were not consistently negative. This has implications for policy and practice, especially for those adhering to the Humanitarian Principles and civil-military global guiding documents which tend to presume this position. This is a significant contribution to the literature, that also clearly indicates the need for further research that systematically examines the perspectives of crisis-affected community members on HMR so as to re-elevate their position as the true nexus of these important and complex debates.

## Data Availability

Data and materials are confidential.

## Footnotes

1 In line with the approach used by recent publications on this issue area—e.g., Grace [18] and Grace et al. [20]—in this manuscript, the term HMR is used so as to disassociate with similar terms used by particular organizations, including humanitarian civil-military coordination (CMCoord, used by the United Nations Office for the Coordination of Humanitarian Affairs; civil-military relations (CMR, used by the International Red Cross and Red Crescent Movement; and civil military cooperation (CIMIC, used by many militaries, among them the North Atlantic Treaty Organization.
